# Prediction of High-Risk Types of Human Papillomaviruses Using Reduced Amino Acid Modes

**DOI:** 10.1155/2020/5325304

**Published:** 2020-06-18

**Authors:** Xinnan Xu, Rui Kong, Xiaoqing Liu, Pingan He, Qi Dai

**Affiliations:** ^1^College of Life Sciences, Zhejiang Sci-Tech University, Hangzhou 310018, China; ^2^College of Sciences, Hangzhou Dianzi University, Hangzhou 310018, China; ^3^College of Sciences, Zhejiang Sci-Tech University, Hangzhou 310018, China

## Abstract

A human papillomavirus type plays an important role in the early diagnosis of cervical cancer. Most of the prediction methods use protein sequence and structure information, but the reduced amino acid modes have not been used until now. In this paper, we introduced the modes of reduced amino acids to predict high-risk HPV. We first reduced 20 amino acids into several nonoverlapping groups and calculated their structure and physicochemical modes for high-risk HPV prediction, which was tested and compared with the existing methods on 68 samples of known HPV types. The experiment result indicates that the proposed method achieved better performance with an accuracy of 96.49%, indicating that the reduced amino acid modes might be used to improve the prediction of high-risk HPV types.

## 1. Introduction

Cervical cancer is a cancer with a higher morbidity and mortality rate among women worldwide [1]. There are about 500,000 new cases of cervical cancer each year, with 280,000 deaths [2], which has become the second largest female cancer [3, 4]. Studies have indicated that human papillomavirus (HPV) infection is closely related to the occurrence and development of cervical cancer, and certain types of HPV cause abnormal tissue growth in the form of papilloma [5–7].

Human papillomavirus belongs to the papillomavirus family. It is an icosahedral, uncoated particle composed of double-stranded DNA of approximately 8,000 nucleotide base pairs [8, 9]. The circular DNA is about 55 nm in diameter [10–13]. To date, there are more than 150 types of human papillomavirus (HPV), and some new HPV types will be found when there are significant homologous differences between some new HPV types and defined HPV types [14–16]. Epidemiological studies have shown a strong correlation between genital HPV and cervical cancer. Genital HPV can be divided into three types according to its relative malignancy: low-risk type, intermediate-risk type, and high-risk type. The clinical association studies usually use two types of HPV: high-risk and low-risk. Low-risk types are associated with low-grade lesions, while high-risk viral types are more closely related to high-grade cervical lesions and cancer [17]. High-risk types included HPV-16, HPV-18, HPV-26, HPV-31, HPV-33, HPV-35, HPV-39, HPV-45, HPV-51-53, HPV-56, HPV-58, HPV-59, HPV-66, HPV-68, HPV-70, HPV-73, HPV-82, and HPV-85 [18]. HPV-16 and HPV-18 accounted for 62.6% and 15.7% of cervical cancers [19], respectively. Therefore, the identification of high-risk HPV has become an important part of the diagnosis and treatment of cervical cancer.

Up to now, many epidemiological and experimental methods can identify HPV types [5, 20–22], mainly using polymerase chain reaction (PCR) technology, and be applied to rapid detection of clinical samples. With the rapid growth of human papillomavirus (HPV) data and sensitivity requirements, we need a reliable and effective calculation method to predict the high-risk types of HPV directly.

In recent years, several computational models have been proposed to predict high-risk HPV types. Eom et al. studied the sequence fragments and introduced genetic algorithms to predict the HPV types [23]. Joung et al. used support vector machines to predict the HPV types based on the hidden Markov model [24, 25]. Park et al. proposed to use decision trees to predict human papillomavirus types [26]. Kim and Zhang calculated the distance of amino acid pairs and further predict the risk types of HPV based on E6 proteins [7, 9]. Kim et al. proposed a set of support vector machines (GSVM) for the classification of HPV types using the differential molecular sequence of protein secondary structure [13]. Esmaeili et al. used ROC to classify HPV types based on Chou's pseudo amino acid composition [27]. Alemi et al. compared the physicochemical properties between the high- and low-risk HPV types, and they used support vector machines to predict the high-risk HPV types [28].

These methods have performed well in the prediction of high-risk HPV types, but the challenge of extracting HPV information remains. The information widely used in the prediction of high-risk types of HPV is based on sequence information, but the information limited to the characteristics of 20 AAs and their reduction groups has not been explored so far. In this paper, we proposed a novel method to predict high-risk types of HPVs based on the reduced amino acid modes. We classified 20 amino acids into several groups and extract their structure and chemical properties. These extracted features were used to predict the high-risk type of HPVs based on a support vector machine. Through some experiments and comparative analysis, we want to evaluate the efficiency of the proposed method, as well as the efficiency of various reduced amino acid modes.

## 2. Materials and Methods

### 2.1. Datasets

There are eight open reading frames that encode early and late genes of the HPVs [11]. The early and late genes have polyA signal 1 and polyA signal 2. The produce of the late genes are L1 and L2 proteins which affect the viral capsid structure [12], while early genes are transformed into E1-E7 proteins. We constructed seven protein databases of the HPVs whose sequences are downloaded from the Los Alamos National Laboratory (LANL). Each protein has 72 HPV types. If a certain type of protein lacks the sequences of HPVs, we downloaded the missing sequence from the National Biotechnology Information Center. Since the E4 protein cannot be found in the National Biotechnology Information Center, its total number is 71. According to an HPV compendium, seventeen HPV types are classified as high-risk types (HPV-16, HPV-18, HPV-31, HPV-33, HPV-35, HPV-39, HPV-45, HPV-51, HPV-52, HPV-56, HPV-58, HPV-59, HPV-61, HPV-66, HPV-67, HPV-68, and HPV-72), and the remaining is low-risk type [13].

### 2.2. Reduced Amino Acids (RedAAs)

20 amino acids have subtle differences, but some of them have similar basic structures and functions. AAindex is a database of physical and biochemical indicators of amino acids established by Tomii and Kanehisa [29]. It mainly includes three parts: AAindex 1, AAindex 2, and AAindex 3. AAindex 1 is a database that describes the physicochemical and biological properties of amino acids. AAindex 2 is the matrix of amino acid mutation, and AAindex 3 is the protein contact potential statistics. These data are from published articles. We mainly used AAindex 1 to calculate the correlation coefficient as the distance between the two indicators. AAindex 1 currently contains 544 indexes, and this article selected 522 indexes. These 522 characteristics are further divided into 7 categories: (A)—alpha and turn propensities, (B)—beta propensity, (C)—composition, (H)—hydrophobicity, (P)—physicochemical properties, and (O)—other properties [29].

Here, we introduced BLOSUM62 to classify amino acids to simplify sequence analysis [30]. We denote the *i*th group as *X*_*i*_ and denote its *j*th amino acid as *X*_*i*_(*j*). Using BLOSUM62, we calculated the similarity score *S*(*X*_*i*_(*j*), *R*_*k*_) between *X*_*i*_(*j*) and the *k*th amino acid *R*_*k*_ as follows:

(1) $$\begin{array}{c} S\left(X_{i}\left(j\right),R_{k}\right)=\text{Blosum}\left(X_{i}\left(j\right),R_{k}\right), \end{array}$$

where Blosum(*X*_*i*_(*j*), *R*_*k*_) denotes the substitution value between *X*_*i*_(*j*) and *R*_*k*_. Then, we summed up all scores of different groups as the score between Seq_*s*_ and Seq_0_:

(2) $$\begin{array}{c} S=\overset{N}{\underset{i=1}{\sum }}\left[\overset{g_{s}\left(i\right)}{\underset{j=1}{\sum }}\overset{g_{0}\left(i\right)}{\underset{k=1}{\sum }}m_{i}\left(k\right)S\left(X_{i}\left(j\right),R_{k}\right)\right]/g_{s}\left(i\right), \end{array}$$

where *g*_0_(*i*) is the *i*th group size of Seq_0_, *g*_*s*_(*i*) is the *i*th group size of Seq_*s*_, *m*_*i*_(*k*) is the total number of *R*_*k*_ occurrences in Seq_0_, and *N* is the group size. *S* measures the degree of retention of parent sequence information. Given a size *N* group, we analyzed all amino acid groups and calculated the similarity score between the parent sequence and the reduced sequence. The reduced alphabets were selected according to their scores. For example, 20 AAs are reduced into 9 RedAAs ({C}, {G}, {P}, {IMLV}, {AST}, {NH}, {YFW}, {DEQ}, and {RK}) in the BLOSUM62 matrix.

### 2.3. Reduced Amino Acid Modes (RedAA Modes)

20 amino acids were divided into the following nonoverlapping groups according to their physicochemical properties in AAindex, and four types of the reduced amino acid modes were calculated as protein structural and physicochemical features.

#### 2.3.1. Content Modes

The first mode is associated with the content-specific features, including the distribution of the RedAA and RedAA pattern in protein sequences.

*(1) K-mer*. Protein sequences and peptides can be seen as a collection of symbols, and their characteristics can be analyzed by the frequency of their small fragments. *k*-mers are *k* consecutive characters in reduced proteins, and a sliding window of length *m* can be used to calculate their frequencies [31–33], moving from position 1 to *m* − *k* + 1 with one base at a time. It allows the overlaps of the *k*-mers and is calculated as

(3) $$\begin{array}{c} f_{w_{\text{RedAA}}}=\frac{{\text{Count}}_{w_{\text{RedAA}}}}{\sum_{x\in R}{\text{Count}}_{x}}, \end{array}$$

where Count_*w*_RedAA__ is the occurrence number of the *k*-mer *w*_RedAA_ and *ℜ* is *k*-mer set of the RedAAs.

*(2) RCTD*. “Composition (C),” “Transition (T),” and “Distribution (D)” are three descriptors of RedAAs, which are defined as follows [34, 35]:

*Composition*: it can be regarded as a single monomer of the reduced sequence, and the sequence components are described by calculating the percentage of each RedAA.

*Transition*: it can be used as the conversion of RedAA *I* and *A* by calculating the frequency of *I* followed by *A*:

(4) $$\begin{array}{c} T_{IA}=\frac{{\text{Count}}_{IA}+{\text{Count}}_{AI}}{N-1}, \end{array}$$

where Count_*IA*_ and Count_*AI*_ are the “*IA*” and “*AI*” numbers, respectively, in the reduced sequence with length *N*.

*Distribution*: it describes the RedAA distribution in the reduced sequence, including the specified coding categories: 25%, 50%, 75%, and 100%.

*(3) PRseAAC*. Type I PRseAAC and type II PRseAAC are widely used pseudoreduced AA compositions (PRseAAC) [36–38].

Type I PRseAAC was proposed by Kuo-Chen Chou, which is defined as follows:

(5) $$\begin{array}{c} \text{PRseAAC}1_{u}=\frac{f_{u}}{\sum_{i=1}^{R}f_{i}+w\sum_{j=1}^{\lambda }\theta_{j}},\;u\leq R,\\ \text{PRseAAC}1_{u}=\frac{w\theta_{u}}{\sum_{i=1}^{R}f_{i}+w\sum_{j=1}^{\lambda }\theta_{j}},\;R\leq u\leq R+\lambda , \end{array}$$

where *f*_*i*_ is the RedAA frequency and *w* is the weighting factor. *θ*_*i*_ is calculated as

(6) $$\begin{array}{c} \theta_{\lambda }=\frac{1}{N-\lambda }\left(\overset{N-\lambda }{\underset{i=1}{\sum }}\Theta \left(R_{i},R_{i+\lambda }\right)\right),\\ \Theta \left(R_{i},R_{j}\right)=\frac{{\left({\text{SH}}_{1}\left(R_{i}\right)-{\text{SH}}_{1}\left(R_{j}\right)\right)}^{2}+{\left({\text{SH}}_{2}\left(R_{i}\right)-{\text{SH}}_{2}\left(R_{j}\right)\right)}^{2}+{\left({\text{SH}}_{3}\left(R_{i}\right)-{\text{SH}}_{3}\left(R_{j}\right)\right)}^{2}}{3},\\ {\text{SH}}_{i}\left({\text{RedAA}}_{i}\right)=\frac{H_{i}\left(\text{RedAA}\right)-\left(\sum_{j=1}^{R}H_{i}\left(j\right)/R\right)}{\sqrt{\sum_{t=1}^{R}{\left(H_{i}\left(t\right)-\left(\sum_{j=1}^{R}H_{i}\left(j\right)/R\right)\right)}^{2}/R}}, \end{array}$$

where *H*_*i*_(RedAA) is the RedAAs' property and *R* is the RedAA size.

Type II PRseAAC can be calculated as

(7) $$\begin{array}{c} \text{PRseAAC}2_{u}=\frac{f_{u}}{\sum_{i=1}^{R}f_{i}+w\sum_{j=1}^{\lambda }\tau_{j}},\;u\leq R,\\ \text{PRseAAC}2_{u}=\frac{w\tau_{u}}{\sum_{i=1}^{R}f_{i}+w\sum_{j=1}^{\lambda }\tau_{j}},\;R\leq u\leq R+\lambda ,\\ \tau_{2\lambda -1}=\frac{1}{N-\lambda }\overset{N-\lambda }{\underset{i=1}{\sum }}H_{i,i+\lambda }^{1},\\ \tau_{2\lambda }=\frac{1}{N-\lambda }\overset{N-\lambda }{\underset{i=1}{\sum }}H_{i,i+\lambda }^{2},\\ H_{i,j}^{1}={\text{SH}}_{1}\left({\text{RedAA}}_{i}\right){\text{SH}}_{1}\left({\text{RedAA}}_{j}\right),\\ H_{i,j}^{2}={\text{SH}}_{2}\left({\text{RedAA}}_{i}\right){\text{SH}}_{2}\left({\text{RedAA}}_{j}\right), \end{array}$$

where *f*_*i*_ is the RedAA frequency, *w* is the weighting factor, SH_*i*_(RedAA) is the RedAAs' property, *R* is the RedAA size, and *N* is the sequence length.

#### 2.3.2. Correlation Mode

The second RedAA mode is based on the characteristics of correlation, which describes the correlation among the RedAAs. In the proposed RedAA mode, three different autocorrelation features are implemented: normalized Moreau–Broto autocorrelation (NMB) [39], Moran autocorrelation (*M*) [40], and Geary autocorrelation (*G*) [41].

*(1) NMB*. The RedAA NMB is defined as

(8) $$\begin{array}{c} \text{NMB}\left(d\right)=\frac{\sum_{i=1}^{N-d}P_{i}^{\text{RedAA}}P_{i+d}^{\text{RedAA}}}{N-d}, \end{array}$$

where *P*_*i*_^RedAA^ denotes the RedAA property at position *i* of the sequence, *d* is the autocorrelation lag, and *N* is the sequence length.

*(2) M*. The RedAA *M* can be calculated as

(9) $$\begin{array}{c} M\left(d\right)=\frac{1/\left(N-d\right)\sum_{i=1}^{N-d}\left(P_{i}^{\text{RedAA}}-{\bar{P}}^{\text{RedAA}}\right)\left(P_{i+d}^{\text{RedAA}}-{\bar{P}}^{\text{RedAA}}\right)}{1/N\sum_{i=1}^{N}{\left(P_{i}^{\text{RedAA}}-{\bar{P}}^{\text{RedAA}}\right)}^{2}},\\ {\bar{P}}^{\text{RedAA}}=\frac{1}{N}\overset{N}{\underset{i=1}{\sum }}P_{i}^{\text{RedAA}}, \end{array}$$

where *P*_*i*_^RedAA^ denotes the RedAA property at position *i* of the sequence, *d* is the autocorrelation lag, and *N* is the sequence length.

*(3) G*. The RedAA *G* is defined as

(10) $$\begin{array}{c} G\left(d\right)=\frac{1/\left(2\left(N-d\right)\right)\sum_{i=1}^{N-d}{\left(P_{i}^{\text{RedAA}}-P_{i+d}^{\text{RedAA}}\right)}^{2}}{1/N\sum_{i=1}^{N}{\left(P_{i}^{\text{RedAA}}-{\bar{P}}^{\text{RedAA}}\right)}^{2}},\\ {\bar{P}}^{\text{RedAA}}=\frac{1}{N}\overset{N}{\underset{i=1}{\sum }}P_{i}^{\text{RedAA}}, \end{array}$$

where *P*_*i*_^RedAA^ denotes the RedAA property at position *i* of the sequence, *d* is the autocorrelation lag, and *N* is the sequence length.

#### 2.3.3. Order Mode

The order mode reflects the physical and chemical interaction among the RedAA pairs. There are two kinds of order modes: sequence coupling score and quasi-sequence score [42].

*(1) Sequence Coupling Score*. The sequence coupling score is calculated:

(11) $$\begin{array}{c} \tau_{d}^{\text{RedAA}}=\overset{N-d}{\underset{i=1}{\sum }}d_{i,i+d}^{\text{RedAA}}, \end{array}$$

where *d*_*i*,*i*+*d*_^RedAA^ is the Schneider-Wrede physicochemical distance or Grantham chemical distance between the RedAAs at positions *i* and *i* + *d* and 1 ≤ *d* ≤ *N*.

*(2) Quasi-Sequence Score*. The quasi-sequence score of the RedAA is defined:

(12) $$\begin{array}{c} \kappa_{\text{RedAA}}=\frac{f_{\text{RedAA}}}{\sum_{i=1}^{R}f_{{\text{RedAA}}_{i}}+w\sum_{d=1}^{M}\tau_{d}^{\text{RedAA}}}, \end{array}$$

where *f*_RAA_*i*__ is the RedAA frequency and *w* denotes the weighting factor.

The quasi-sequence score can be calculated as

(13) $$\begin{array}{c} \kappa_{\tau }=\frac{w\tau_{d}^{\text{RedAA}}}{\sum_{i=1}^{R}f_{{\text{RedAA}}_{i}}+w\sum_{d=1}^{M}\tau_{d}^{\text{RedAA}}}, \end{array}$$

where *τ* is the sequence coupling score, *f*_RAA_*i*__ is the RedAA frequency, and *w* denotes the weighting factor.

#### 2.3.4. Position Mode

The position mode represents the distribution of RedAA positions of protein sequences based on the coefficient of variations [32, 43]. First, we converted the protein sequence into a digital sequence *N*(RedAA) and calculated the probabilities *P*_RedAA_(*ξ*) of the separation distance *ζ* between two adjacent RedAAs. The mean *E*_(RedAA)_(*ξ*) and variance *D*_(RedAA)_(*ξ*) are defined:

(14) $$\begin{array}{c} E_{\left(\text{RedAA}\right)}\left(\xi \right)=\underset{\xi }{\sum }\xi \times P_{\left(\text{RedAA}\right)}\left(\xi \right),\\ D_{\left(\text{RedAA}\right)}\left(\xi \right)=E_{\left(\text{RedAA}\right)}\left(\xi^{2}\right)-{\left[E_{\left(\text{RedAA}\right)}\left(\xi \right)\right]}^{2}. \end{array}$$

We then calculated the positional information *C*_(RedAA)_(*ξ*):

(15) $$\begin{array}{c} C_{\left(\text{RedAA}\right)}\left(\xi \right)=\frac{E_{\left(\text{RedAA}\right)}\left(\xi \right)}{\sqrt{D_{\left(\text{RedAA}\right)}\left(\xi \right)}}, \end{array}$$

where *C*_(RedAA)_(*ξ*) is the reciprocal of the coefficient of variation (CV) which compares the degree of change between two datasets, even if there are large differences between their means. In this paper, it was denoted as the RedAA position characteristics.

### 2.4. Prediction Algorithm

*Y* = [*y*_1_,  *y*_2_,  ⋯, *y*_*n*_]^*T*^ is an HPV label set, *y*_*i*_ = 1 is from the high-risk type, and *y*_*i*_ = 2 is from the low-risk type. We used *x*_*ij*_ to represent the *j*th features of the RedAA modes of the *i*th HPV sample, where *j* = 1, 2, ⋯, *m*. All of the features of the RedAA modes for all HPV samples are denoted as

(16) $$\begin{array}{c} X=\begin{array}{c} x_{1}\\ x_{2}\\ \vdots \\ x_{n} \end{array}\overset{\text{index}1\text{ index}2\;\cdots \text{ index}m}{\left[\begin{array}{cccc} x_{11} & x_{12} & \cdots & x_{1m}\\ x_{21} & x_{22} & \cdots & x_{2n}\\ \vdots & \vdots & \ddots & \vdots \\ x_{n1} & x_{n2} & \cdots & x_{nm} \end{array}\right]}. \end{array}$$

We used a support vector machine (SVM) to predict the HPV type, which is expressed as follows:

(17) $$\begin{array}{c} \underset{w,b,\xi }{\min}\;J\left(w,b,\xi \right)=\frac{1}{2}\left(w^{T}w\right)+C\overset{n}{\underset{i=1}{\sum }}\xi_{i}\\ \text{subject to}\;\left\{\begin{array}{cc} y_{i}\left[w^{T}\varphi \left(x_{i}\right)+b\right]\geq 1-\xi_{i}, & i=1,2,\cdots ,n,\\ \xi_{i}\geq 0, & i=1,2,\cdots ,n, \end{array}\right. \end{array}$$

where *w* is a linear combination of a set of nonlinear data conversion:

(18) $$\begin{array}{c} w=\overset{n}{\underset{i=1}{\sum }}\alpha_{i}y_{i}\varphi \left(x_{i}\right), \end{array}$$

where *b* denotes the bias term, *C* denotes some regularization parameters, and *ξ*_*i*_ is the training error. The above problem can be expressed:

(19) $$\begin{array}{c} \underset{\alpha }{\max}\;J\left(\alpha \right)=\underset{\alpha }{\max}\overset{n}{\underset{i=1}{\sum }}\alpha_{i}-\frac{1}{2}\overset{n}{\underset{i=1}{\sum }}\overset{n}{\underset{j=1}{\sum }}\alpha_{i}\alpha_{j}y_{i}y_{j}\varphi {\left(x_{i}\right)}^{T}\varphi \left(x_{j}\right)\\ \text{subject to}\;\left\{\begin{array}{cc} \overset{n}{\underset{i-1}{\sum }}\alpha_{i}y_{i}=0, & i=1,2,\cdots ,n,\\ 0\leq \alpha_{i}\leq \text{C}, & i=1,2,\cdots ,n. \end{array}\right. \end{array}$$

Here, the Gaussian kernel function is used to calculate *φ*(*x*_*i*_)^*T*^*φ*(*x*_*j*_) instead of *φ*(*x*_*i*_) and *φ*(*x*_*j*_). The separation problem can be expressed:

(20) $$\begin{array}{c} f\left(x\right)=\overset{n}{\underset{i=1}{\sum }}\alpha_{i}y_{i}K\left(x_{i},\text{x}\right)+b,\\ y\left(x\right)=\mathrm{sign}\left[f\left(x\right)\right]. \end{array}$$

The training model can predict the risk type of the test sample *x* ∈ *R*^*m*^ according to the following formula:

(21) $$\begin{array}{c} y\left(x\right)=\left\{\begin{array}{cc} 1, & \text{if }f\left(x\right)>0,\\ 2, & \text{if }f\left(x\right)\leq 0. \end{array}\right. \end{array}$$

*y*(*x*) = 1 indicates that the sample *x* belongs to the high-risk type; otherwise, it belongs to the low-risk type. In order to obtain a better model, we used a simple grid search strategy based on 10-fold cross-validation to find the optimal model for each dataset.

## 3. Results and Discussion

### 3.1. Evaluation Measures

There are three popular methods to evaluate the efficiency of prediction models: subsampling test, independent test, and jackknife test. Since the jackknife test can evaluate the efficiency of various predictor variables, we used it to evaluate the efficiency of the proposed method and calculated the class accuracies and overall accuracies:

(22) $$\begin{array}{c} \text{specificity}\left(\text{accuracy of high}‐\text{risk type}\right)=\frac{a}{a+c},\\ \text{sensitivity}\left(\text{accuracy of low}‐\text{risk type}\right)=\frac{d}{b+d},\\ \text{accuracy of totality}=\frac{a+d}{a+b+c+d}\cdot 100\%, \end{array}$$

where *a* denotes true positives, *c* denotes false positives, *d* denotes true negatives, and *b* denotes false negatives.

### 3.2. HPV Classification

We used the jackknife test to evaluate the performance of the proposed RedAA modes. We divided the 20 amino acids into 5 to 19 groups and calculated their RedAA modes as protein features and then input them into the support vector machine to predict the HPV type.  Table 1 shows the tagged HPV types and the predicted results.

It can be seen from Table 1 that the 65 HPV types predicted by our method are consistent with the actual types and have better performance. However, HPV-72 is predicted to be low-risk but is actually high-risk, and HPV-30 is predicted to be high-risk but is actually low-risk. For further comparison, we compared our results with Kim et al.'s results [13]. For Kim et al.'s prediction, HPV-56 was predicted to be potentially high-risk, and we predicted it to be high-risk; HPV-53 and HPV-73 were predicted to be potentially high-risk, but in our results, they were low-risk. Phylogenetic analysis showed that HPV-30 was closely related to the established oncogenic type HPV-56, suggesting that HPV-30 was more likely to be a high-risk type. The results show that the proposed method is more consistent with the actual risk type.

We further compared our method with the following method: SVM based on the mismatch [24], SVM classifier based on the linear kernel [13], SVM based on the gap spectral kernel (Gap) [7], BLAST model [13] and integrated SVM (Ensemble) [13], and two text prediction methods based on AdaCost [26] and naive Bayes [26]. The accuracy of our method reaches 96.49%, while the accuracy of the integrated SVM is 94.12%, the accuracy of the SVM based on the unmatched kernel is 92.70%, the accuracy of the SVM based on the linear kernel is 90.28%, and the accuracy of BLAST reaches 91.18%. As for the text prediction method, AdaCost [26] has an accuracy rate of 93.05%, while naive Bayes [26] has an accuracy rate of 81.94%. The comparison also shows that the RedAA model is more effective in classifying the risk types of human papillomaviruses.

### 3.3. The Performance of the Early and Late Proteins in HPV Type Prediction

**Figure 1:**
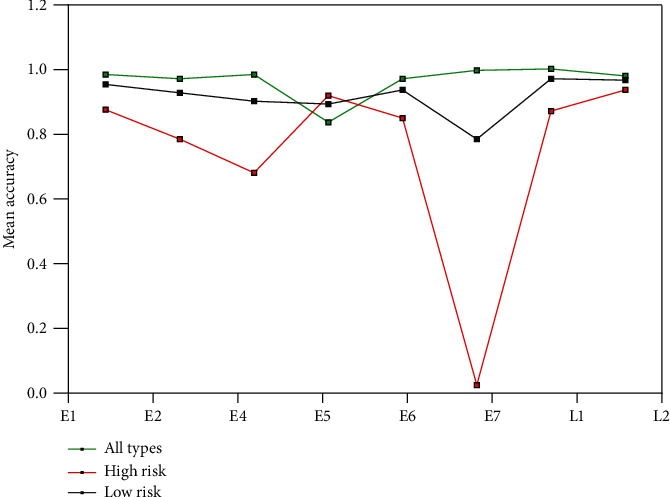
Comparison of prediction accuracy of each class based on all the early and late proteins.

Early HPV proteins contain E1, E2, E4, E5, E6, and E7, and late proteins include L1 and L2 [3, 5]. Information commonly used for high-risk and low-risk HPV prediction includes information on protein sequences, secondary structure, and pseudoamino acid composition, in which most of them use E6, E7, or L1 protein [23–28]. In this paper, we used seven protein datasets of early and late proteins in HPV type prediction and compared their performance.  Figure 1 compares the accuracy of each category and the overall accuracy based on early and late proteins.

Figure 1 shows that the prediction accuracy of low-risk types is higher than that of high-risk types, except for E5 protein. L1 protein outperforms other HPV proteins in the prediction of low-risk types. L2 protein performs best in high-risk type predictions. The above research shows that E6, E7, L1, and L2 proteins are closely related to high-risk HPV and play an important role in the occurrence and development of diseases [14]. The function of L1 protein in low-risk and high-risk types is not exactly the same. L1 protein in the high-risk type exists in the form of integration, and L1 gene product self-assembly efficiency is low. L1 protein in the low-risk type exists in the form of free tissue, with high self-assembly efficiency. In high-risk typing, if L1 protein mutates, L1 protein cannot combine with L2 protein to form capsid protein and then cannot assemble HPV-infected virus particles. When HPV enters the host cell, the viral DNA replicates in large quantities and can integrate with the host cell DNA, resulting in host cell infection, infinite value addition, and cell immortalization. The results show that L1 protein performs better in the prediction of high-risk HPV types, while L2 protein is more suitable for low-risk HPV types.

### 3.4. Influence of the Physicochemical Properties of Amino Acids

**Figure 2:**
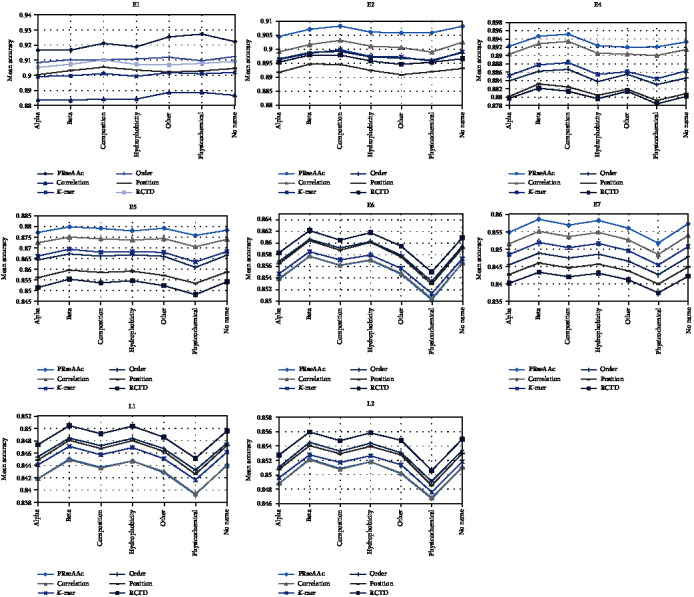
Comparison of the mean of the overall accuracies of HPV type prediction based on seven physicochemical property classes and six RedAA modes for all the early and late proteins.

The proposed method reduced 20 AAs into several nonoverlapping groups, which relies heavily on the physical and biochemical indices of amino acids. The 522 characteristics of AAindex are divided into seven categories according to their physical and biochemical features [29]. The largest group is hydrophobicity and the second largest group is alpha and turn propensities, and the sizes of the other four groups are relatively small. For each HPV protein, we used 522 physicochemical properties to calculate six kinds of reduced AA modes. For each class of the physicochemical properties of amino acids, we calculated their mean of the overall accuracies of HPV type prediction. The comparison of different physicochemical property classes and the RedAA modes is shown in Figure 2.

From Figure 2, it can be found that the proposed prediction has no obvious preference among 7 classes of physicochemical properties for E1 proteins. As for E2 proteins, composition is the best of the six reduced AA modes. For E4 proteins, the physicochemical properties of beta and composition are better. For the reduced AA mode position and RCTD, the physicochemical properties of beta are better in prediction, but composition is better for the other four modes. The results of E5, E6, E7, L1, and L2 proteins are similar to those of E2 proteins, and the six reduced AA modes show better performance in beta physicochemical properties. These results indicate that E5, E6, E7, L1, and L2 proteins have a preference for beta physicochemical properties to reduce amino acids and calculate the six reduced AA modes in HPV type prediction.

### 3.5. Comparison of the Reduced Amino Acid Modes

In order to evaluate the performance of different modes, we used 522 physicochemical properties to calculate the RedAA modes of all the early and late proteins and calculated their average of the overall accuracies of HPV type prediction, which is shown in Figure 2. Figure 2 shows that six RedAA modes have the same preference trend among seven classifications of the physicochemical properties. As for E1, E2, E4, E5, and E7 proteins, PRseAAC is better than the other RedAA modes, and the average accuracy of its prediction of HPV typing is also significantly higher than the average of other RedAA modes. As for E6, L1, and L2 proteins, RTCD outperforms the other five RedAA modes. In addition, PRseAAC and RTCD show better performance in beta physicochemical properties of the amino acids.

### 3.6. Influence of the Number of Reduced Amino Acids

**Figure 3:**
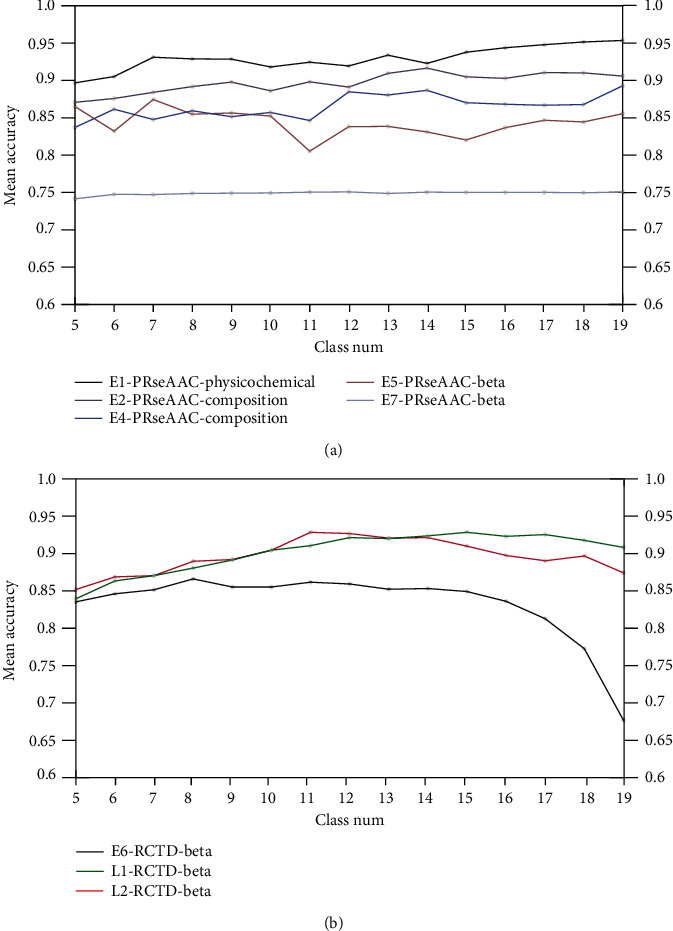
Performance comparison of the RedAA modes PRseAAC and RTCD with different reduced amino acids: (a) the average accuracies of the PRseAAC and RTCD with 5-19 reduced amino acids for E1, E2, E4, E5, and E7 and (b) the average accuracies of the PRseAAC and RTCD with 5-19 reduced amino acids for E6, L1, and L2.

The proposed method used the structural and physicochemical features of reduced amino acids, which reduces the dimension of input information and improves the efficiency of the prediction model. However, it should be noted that the RedAA modes are associated with the number of reduced amino acids. In order to discuss the influence of the RedAA size, we reduced 20 amino acids into 5-19 classes based on 522 physicochemical properties and calculated their RedAA modes PRseAAC and RTCD for of all the early and late proteins. The average accuracies of the RedAA modes PRseAAC and RTCD with 5-19 RedAAs are summarized in Figure 3.

Figure 3 shows the accuracy of HPV type prediction with the increase in reduced amino acids when combining the PRseAAC and physicochemical properties of amino acids for E1 proteins, and the best-performing PRseAAC achieves 95.378% accuracy with 19 reduced amino acids. For E2 proteins, the prediction model achieves the best performance with the PRseAAC and the physical and physicochemical properties of the composition class when amino acids are reduced to 14 classes. As for E5 and E7, PRseAAC achieves 87.18% and 75.07% accuracies when 20 amino acids are reduced to 7 and 12 classes, respectively. For E6, L1, and L2 proteins, the combination of the RCTD and beta physicochemical properties achieves best performances with 8, 15, and 11 reduced amino acids, respectively.

## 4. Conclusion

Genital papillomavirus is closely related to cervical cancer, especially high-risk HPV. Therefore, the identification of the HPV risk type is of great significance for the cervical cancer. We proposed a computational method for the prediction of the high-risk HPV based on the RedAA modes. With the help of the physicochemical properties of the amino acids, we reduced 20 amino acids into several nonoverlapping groups and calculated the structure and physicochemical characteristics of reduced AAs (RedAA) as the RedAA modes. We used reduced sequence information to predict high-risk types of HPV. Experiments with 68 known HPV types show that the proposed method has better performance than previous methods.

The first contribution is that L1 protein performs better in the prediction of high-risk HPV types, while L2 protein is more suitable for low-risk HPV types. The second contribution can be indicated from the influence of the physicochemical properties of amino acids; we noticed that E5, E6, E7, L1, and L2 proteins have a preference for beta physicochemical properties to reduce amino acids. The third contribution can be deduced from the comparison of the reduced amino acid modes; we found that the PRseAAC and RTCD outperform the other four RedAA modes and show better performance in beta physicochemical properties of the amino acids. The final contribution can be seen from the influence of the number of reduced amino acids; we noticed that the combination of the RCTD and beta physicochemical properties achieves the best performances with 8, 15, and 11 reduced amino acids for E6, L1, and L2 proteins, respectively.

## Figures and Tables

**Table 1:** Comparison of the real risk types (REAL) and the prediction results using the proposed approach.

Types	Real	Predicted	Types	Real	Predicted	Types	Real	Predicted	Types	Real	Predicted
HPV-39	High	High	HPV-7	Low	Low	HPV-34	Low	Low	HPV-50	Low	Low
HPV-72	High	Low	HPV-30	Low	High	HPV-44	Low	Low	HPV-5	Low	Low
HPV-33	High	High	HPV-73	Low	Low	HPV-43	Low	Low	HPV-20	Low	Low
HPV-51	High	High	HPV-6	Low	Low	HPV-32	Low	Low	HPV-23	Low	Low
HPV-16	High	High	HPV-27	Low	Low	HPV-24	Low	Low	HPV-19	Low	Low
HPV-56	High	High	HPV-13	Low	Low	HPV-8	Low	Low	HPV-47	Low	Low
HPV-18	High	High	HPV-55	Low	Low	HPV-48	Low	Low	HPV-22	Low	Low
HPV-59	High	High	HPV-2	Low	Low	HPV-12	Low	Low	HPV-25	Low	Low
HPV-52	High	High	HPV-10	Low	Low	HPV-49	Low	Low	HPV-9	Low	Low
HPV-35	High	High	HPV-42	Low	Low	HPV-15	Low	Low	HPV-36	Low	Low
HPV-68	High	High	HPV-28	Low	Low	HPV-21	Low	Low	HPV-41	Low	Low
HPV-58	High	High	HPV-40	Low	Low	HPV-4	Low	Low	HPV-63	Low	Low
HPV-31	High	High	HPV-3	Low	Low	HPV-65	Low	Low	HPV-1	Low	Low
HPV-66	High	High	HPV-11	Low	Low	HPV-37	Low	Low	HPV-80	Low	Low
HPV-45	High	High	HPV-29	Low	Low	HPV-38	Low	Low	HPV-77	Low	Low
HPV-61	High	High	HPV-74	Low	Low	HPV-60	Low	Low	HPV-76	Low	Low
HPV-67	High	High	HPV-53	Low	Low	HPV-17	Low	Low	HPV-75	Low	Low

## Data Availability

All the data used to support the findings of this study are available from the Los Alamos National Laboratory (https://pave.niaid.nih.gov/lanl-archives).
